# A 30 mm sized gastrojejunostomy may lead to a lower rate of therapy failure in comparison to a 45 mm sized gastrojejunostomy following laparoscopic Roux-en-Y gastric bypass

**DOI:** 10.1016/j.amsu.2022.103787

**Published:** 2022-05-16

**Authors:** Oliver Stumpf, Volker Lange, Anke Rosenthal, Rolf Lefering, Christoph Paasch

**Affiliations:** a Center of Obesity and Metabolic Surgery, Helios Klinikum Berlin-Buch, Schwanebecker Chaussee 50, 13125, Berlin, Berlin, Germany; b Obesity Outpatient Clinic, Bismarckstraße 101, 10625, Berlin, Germany; c IFOM-Institute for Research in Operative Medicine, University Witten/Herdecke, Faculty of Health, Cologne, Germany; d Department of General Surgery, University Hospital Brandenburg an der Havel, Hochstraße 29, 14770, Brandenburg an der Havel, Germany; e Faculty of Medicine, University Hospital Brandenburg, Brandenburg Medical School Theodor Fontane, Brandenburg, Germany

**Keywords:** Bariatric surgery, Gastroenterostomy, Gastrojejunostomy, Gastric bypass, Morbid obesity, Weight loss

## Abstract

**Background:**

In bariatric surgery the laparoscopic Roux-en-Y gastric bypass (LRYGB) has been proven to be a safe and effective approach. Currently the optimal size of the linear-stapled gastrojejunostomy (GJ) and its impact on weight loss are not known due to a lack of clinical trials on that topic. We aimed to provide evidence on the impact of the GJ size in terms of gastric bypass weight loss.

**Methods:**

Patients who underwent LRYGB due to morbid obesity were retrospectively analyzed from January 2013 to January 2016. While the procedure was completely standardized, one surgeon continued using the 45 mm sized linear stapler to perform GJ while the other switched to using a 30 mm cartridge.

**Results:**

277 patients were female (78%) and 77 males. The average age was 41.7 ± 12.3 years. In 118 cases a 30 mm sized GJ was conducted. 236 individuals received a 45 mm sized GJ. In terms of gender, age, length of biliary and alimentary limb both groups were homogenous. Individuals with a 30 mm sized GJ had a statistical significant lower rate of therapy failure (Excess weight loss <25%, 25–49%, ≥50% after 3 years, P value χ^2^ for trend <0.035).

The excess weight loss did not significant differ between both groups.

**Conclusions:**

A 30 mm sized GJ may lead to a lower rate of therapy failure in comparison to a 45 mm sized GJ following laparoscopic Roux-en-Y gastric bypass. Prospective trials are mandatory to confirm our findings.

## Highlights

• Currently the optimal size of the GJ, when performing a LRYGB, and its impact on weight loss are not well known due to a lack trial.
• A 30 mm sized gastrojejunostomy (GJ) may lead to a lower rate of therapy failure in comparison to a 45 mm sized gastrojejunostomy.
• Only patients (n = 4) in the 45 mm GJ group reported a weight gain after three years.

## 1 Introduction

The prevalence of morbid obesity is increasing globally [1]. In the USA approximately one third of the adults was considered to be obese in 2010 [2]. According to the World Health Organization (WHO) more than 1.9 billion adults (≥18 years) suffered from obesity in 2014 [3].

Overweight and obesity are associated with an increased risk of morbidity and mortality. Due to limited success of nonsurgical weight loss strategies the role and demand of bariatric surgery for morbid obesity is increasing [2]. Several surgical techniques including LRYGB have been proven to provide sufficient weight loss and reduction of obesity-related comorbidities like type II Diabetes and Hypertension [[4], [5], [6], [7], [8], [9]].

Currently the optimal size of the GJ, when performing a LRYGB, and its impact on weight loss are not well known due to a lack of trial results [10]. Heneghan et al. considered a gastrojejunal stoma >2 cm enlarged and have shown the inverse relation between pouch/stoma size and weight regain [11]. But because of the known impact of banding of the pouch on weight loss it is hypothesized that a smaller GJ may increase the extent of gastric bypass induced weight loss [12,13]. To that, Lemmens conducted a retrospective analysis among 432 patients who underwent RYGB. In 178 cases a banded-GaBP Ring was used.

The author demonstrated that these rings prevented further weight regain in the majority of cases [14]. The study at hand aimed to provide more evidence on the impact of the GJ size in terms gastric bypass weight loss and therapy success rate.

## 2 Methods

A mono-centre retrospective analysis of individuals, who underwent LRYGB due to morbid obesity was conducted from January 2013 to December 2019.

The study has been performed at the Department of Bariatric Surgery, Vivantes Klinikum Berlin (Germany). The study was approved by the Ethics Committee of the Ärztekammer Berlin (Medical Association Berlin) in October 2019 (Eth-17/19) and conducted in accordance with the ethical standards of the Helsinki Declaration of 1975.

The study was registered with the German clinical trial registry DRKS (DRKS00019016). No funding has been received. The study has been performed according to the STROCCS guidelines [15].

The study is exclusively based on data available from the patients’ files. The patients were followed in outpatient basis repeatedly up to 4 years postoperatively on a regular basis. During this period the information has been considered from the patient files. There was no separate contact to the patients for this study. No informed consent was needed.

The enrolled patients underwent a standard preoperative preparation including clinical, psychological and multidisciplinary evaluation. A gastroscopy including endoscopic biopsy to detect a potential *Helicobacter pylori* infection, general laboratory tests, electrocardiogram and a pulmonary function test was conducted in all patients.

### 2.1 Inclusion and exclusion criteria

All patients who underwent LRYGB as a primary procedure due to morbid obesity have been included. The morbid obesity was defined according to the German guidelines (from 2018) [16].

Patients under the age of 18 have been excluded. In addition, 9 patients with no follow-up investigation, and further 15 cases with only a short-term follow-up (less than 6 months) were excluded. This left 354 patients for analysis.

### 2.2 Surgical approach

The LRYGB was conducted according to the published approach by Wittgrove et al. (1997) either with a 30 mm or a 45 mm sized stapled GJ [7,17,18].

The patients were positioned in an open-legs reverse-Trendelenburg position. The Pneumoperitoneum was set by placing a vision controlled optical trocar paramedian left between umbilicus and epigastric angle. A total of 4 trocars and a liver retractor were placed. The operation was started with conducting the gastric pouch, starting with dissection of the esophagogastric angle and removal of the fat pad. The lesser curvature dissection was performed with ultrasonic ligation below the second pair of gastric vessels, creating a 6 cm long pouch and getting access to the posterior gastric wall. The first firing of the linear cutter stapler (Medtronic, USA or Ethicon, USA) was performed in horizontal guidance of 35 mm of a 45 mm cartridge (blue cartridge). The creation of the longitudinal pouch was then continued in a standardized fashion vertically to reach 0,5 cm lateral of His parallel to the lesser curvature, thus creating a longitudinal cylinder. The numbers of cartridges depended on the thickness of the gastric wall tissue as well as the size of cartridge chosen (45 or 60). At times an additional cartridge was used for remaining tissue exceeding the capacity for a clip. The pouch volumes are estimates of the operating surgeons and were taken from the original surgery reports.

The duodenojejunal angle and initial segment of the jejunum were identified in order to define a length of 80 cm for the biliopancreatic limb. In cases of a BMI <40 kg/m^2^ the biliopancreatic limb with 70 cm length was created whereas patients received a 100 cm biliopancreatic limb when the BMI was >60 kg/m^2^. Keeping the proximal portion of the jejunum always to the left side of the patient, the loop was moved to the upper abdomen without division. If needed due to mesenteric tension, the greater omentum was partially vertically divided in its middle portion. After making two small enterotomies with ultrasonic scissors, one antimesenteric at the measured jejunal loop wall and another on the latero-posterior face of the gastric pouch, the GJ was performed by linear stapler with 45-mm or 30-mm cartridge (blue) for the posterior part and completed by 3-0 monofilament resorbable running suture ventrally. Then, the alimentary limb was separated from the afferent loop by 45 mm cartridge (white) close to the GJ and Methylen-Blue-Test was performed. From the GJ, 135 cm of jejunum was measured, defining the length of the alimentary limb and an entero-enterostomy was done with the distal biliopancreatic limb. In cases of a BMI <40 kg/m^2^ the alimentary limb with 125 cm length was created whereas patients received a 150 cm alimentary limb when the BMI was >60 kg/m^2^. The anastomosis was conducted anisoperistaltic either by three staple techniques (white cartridge) or using a 2 staple technique and closure by 3-0 monofilament resorbable suture. The mesenteric space was not routinely closed as we do now. We did not place drainages routinely.

### 2.3 Definitions

For each patient the normal weight has been calculated as an equivalent to a BMI. The excess weight was calculated as the difference between the pre-operative weight and the weight at BMI of 25 kg/m^2^. Weight loss after surgery (EWL, excess weight loss) was then calculated as a percentage of this excess weight.

The primary endpoint was EWL after 3 years.

The secondary endpoints were EWL at 1 and 2 years, the percentage of patients with less than 25% or 50% EWL and postoperative complications according to the Clavien-Dindo-Classification (CD) [19].

### 2.4 Statistics

Since time points of follow-up sometimes were not exactly at 12, 24, or 36 months, linear interpolation was used to estimate the body weight at these exact time points. Not all patients have had complete data at all follow-up time points. One year data were available from 276 cases (78%); two years data were available from 246 cases (70%), and three years data were available from 124 cases (35%). Missing weight data were interpolated. Since weight loss occurred mainly in the first year, linear interpolation was not used in case of missing first year data. Instead, the first available weight from later follow-up was used to impute the missing weight at one year. If no later follow-up was available for interpolation, weight changes were assumed to follow the same trend observed in patients who had the respective data: patients with a 30 mm sized GJ showed a decrease of 7.0% from year 1–2, and an increase of 1.3% from year 2–3. Patients with a 45 mm sized GJ showed a decrease of 5.4% and an increase of 2.1%, respectively.

Patient groups who received a 30 mm or a 45 mm sized GJ were compared using descriptive statistics. Categorical measures were presented as counts with percentage; continuous measures were presented as mean with standard deviation (SD), or median with inter-quartile range. Fisher's exact test and Mann-Whitney *U* test were used for categorical and continuous measurements, respectively. Categories of EWL were compared using chi-squared test for trend. A *p*-value <0.05 was considered statistically significant.

Selected findings were presented with a 95% confidence interval (95% CI). The analysis has been performed using SPSS statistical software (version 26; IBM Inc., Armonk, NY, USA) and R (version 3.6.2).

To calculate an adjusted effect for the effect of 30/45 mm anastomosis, multiple linear regression analysis was performed. The outcome measure (or dependent variable) was absolute weight loss after 2 years. The following variables were used as independent predictors: Preoperative weight (kg), Age (years), young patients (<30 years), gender, diabetes, hypertension, biliary limb (>80 cm), alimentary limb (>135 cm), pouch volume >15 ml, postoperative complication (CD > 0) and size of anastomosis.

## 4 Results

**Fig. 1:**
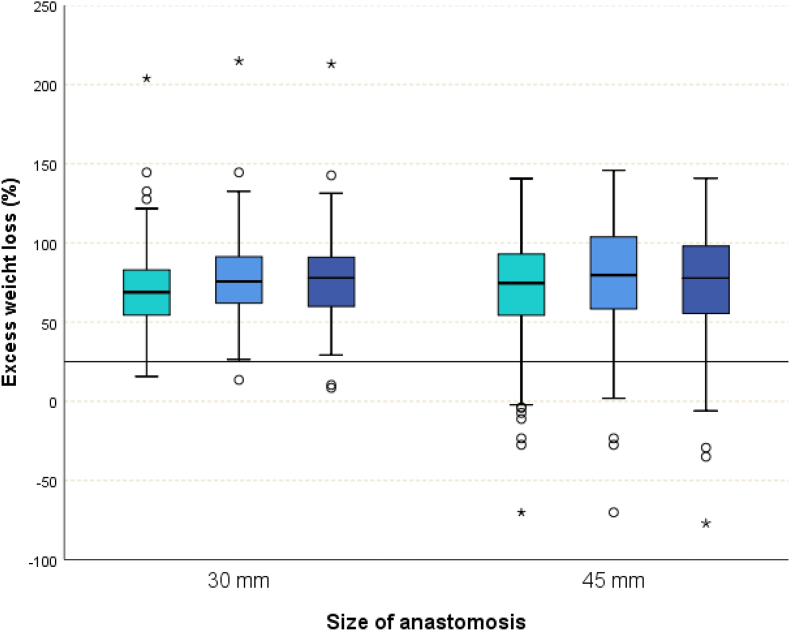
Box plot of excess weight loss (%) after 1 (pale blue), 2 (blue), and 3 (dark blue) years in both study groups. (For interpretation of the references to colour in this figure legend, the reader is referred to the Web version of this article.)

354 patients who underwent RYGB were analyzed. The initial weight and gastric bypass weight loss for both groups are depicted in Fig. 1.

### 4.1 Univariate analysis on baseline characteristics

The average age was 41.7 ± 12.3 years. The majority of patients were female (n = 277, 78.2%). The average BMI was 45.6 ± 7.0 kg/m^2^. In 118 cases a 30 mm sized GJ was conducted (33.3%) while the remaining 236 individuals received a 45 mm sized GJ (Table 1). No conversion to open surgery was observed. Detailed information is provided in Table 1.

**Table 1:** Basic descriptive data of patients with a 30 mm and 45 mm sized GJ.

	30 mm size n = 118	45 mm size n = 236	all patients n = 354	p-value
Age (years)	41.8 (13.3)	41.7 (11.7)	41.7 (12.3)	.998
Females Males	89 (75.4%) 29 (24.6%)	188 (79.7) 48 (20.3%)	277 (78.2%) 77 (21.8%)	.412
Body weight before surgery (kg)	137 (26)	127 (23)	130 (24)	<.001
BMI before surgery	47.5 (7.5)	44.6 (6.5)	45.6 (7.0)	<.001
Pouch volume (ml)	14.9 (1.0)	15.3 (1.4)	15.2 (1.3)	.007
Biliary limb (cm)	82 (9)	80 (9)	81 (9)	.122
Alimentary limb (cm)	139 (9)	141 (9)	140 (9)	.082
Complications acc. to Clavien-Dindo				
I	0	1	1	.867
II	1	3	4	
III	2	3	5	
IV/V	0	0	0	

Postoperative complications consisted of bleeding (occurrence <30 days), anastomotic leaks (only among patients with a 45 mm sized GJ, stent placement took place) and ulcera (occurrence >30 days). The follow-up did not detect any postoperative fistulas in both groups. No CD grade IV and V occurred among all patients (Table 1).

In terms of age, gender, biliary and alimentary limb size and postoperative complications no statistical significance was detected between both groups. In terms of BMI at operation (*p* < 0.001) and the size pouch volume (*p* = 0.007) the groups differ statistical from each other.

### 4.2 Univariate analysis on TWL

The findings of TWL analysis are depicted in Table 2.

**Table 2:** Univariate analysis on excess and total weight loss after 1, 2 and 3 years following surgery.

TWL and EWL	1 year	2 years	3 years
30 mm GJ (n = 118)			
TWL in kg (median, IQR)	44 [32–56]	48 [36–61]	46 [36–60]
EWL in % (median, IQR)	69 [54–83]	76 [62–91]	78 [60–91]
EWL categories			
weight gain (n)^a^	0	0	0
<25%	2 (1.7%)	1 (0.8%)	2 (1.7%)
25–49%	16 (13.6%)	13 (11.0%)	13 (11.0%)
≥50%	100 (84.7%)	104 (88.1%)	103 (87.3%)
45 mm GJ (n = 236)			
TWL in kg (median, IQR)	37 [29–49]	42 [31–53]	41 [30–51]
EWL in % (median, IQR)	75 [54–93]	80 [58–104]	78 [55–98]
EWL categories			
weight gain (n)^a^	8	3	4
<25%	17 (7.2%)	15 (6.4%)	17 (7.2%)
25–49%	33 (14.0%)	25 (10.6%)	30 (12.7%)
≥50%	186 (78.8%)	196 (83.1%)	189 (80.1%)
P value (χ^2^ for trend)	0.061	0.060	**0.035**

GJ gastro-jejunostomy, TWL total weight loss, EWL excess weight loss, IQR inter-quartile range.

a Weight gain as part of the patients collective EWL <25.

### 4.3 Univariate analysis on EWL after three years

Individuals who received a 30 mm GJ had an EWL of 78% (IQR 60–91). Patients with a 45 mm sized GJ had an EWL of 78% (IQR 55–98). No statistical significance was detected (*p* > 0.001; Fig. 1).

### 4.4 Univariate analysis on EWL categories

The univariate analysis is summarized in Table 2. In terms of all EWL categories (<25%, 25–49%, ≥50%) a statistical significance was revealed between the two groups (P value χ^2^ for trend <0.035) favoring the 30 mm sized GJ (Table 2, Fig. 1).

### 4.5 Multivariate regression

The following predictors had no or only a minor influence in the model (p > 0.30): Hypertension, alimentary limb (>135 cm), pouch volume (>15 ml), postoperative complications (CD > 0). The size of anastomosis (30 mm) only caused an additional weight loss of 0.38 kg (p = 0,82; 95% confidence interval −3.04–3.80). Overall, an r of 0.716 and an r^2^ of 0.513 was achieved in this model. Gender, young age, diabetes, and preoperative weight were identified as independent predictors of absolute weight loss after 2 years (Table 3).

**Table 3:** Result of multivariable linear regression analysis with absolute weight loss two years after GJ as dependent variable; thus the coefficients are the adjusted weight loss in kg. Predictors with only marginal effect (p > 0.30) except size of anastomosis, were not presented here.

	Regression coefficient	Standard error	P value
Preoperative weight (per kg BW)	0,69	0.04	<0.001
Young age (<30 years)	3,98	2.05	0.053
Female	15,56	2.16	<0.001
Biliary limb >80 cm	−3,82	2.76	0.167
Diabetes	−3,55	1.79	0.048
GJ anastomosis size 30 mm	0,38	1.71	0.824
Constant	−55,85	6.35	<.001

BW – body weight; GJ gastro-jejunostomy.

## 5 Discussion

The LRYGB has first been described by Mason and Ito in 1967 [7]. The approach was further modified by Wittgrove et al. [17,18] and is today considered as a feasible approach to treat morbid obesity [2,4,6].

Due to lack of evidence it remains unclear, which GJ size should be chosen to prevent GJ-related complications and to achieve a sufficient EWL [2,4,6].

On the one hand, smaller GJs may facilitate a better EWL than larger anastomoses [[20], [21], [22], [23]]. It has been assumed by some authors, that a greater aliment restriction in a smaller GJ may lead to a less volumetric capacity and stronger hunger control [10]. Furthermore, a slow gastric pouch emptying may lead to lasting satiety, less hunger in between meals, and prolonged production of gastric hormones resulting in more incretinic effect [10]. To that, when performing a retrospective analysis on patients (n = 128), who received either a 15 mm or 45 mm sized GJ, Ramos et al. demonstrated, that the individuals with a small GJ lost statistical significant more weight after two years [10]. We did not reveal a more sufficient postoperative EWL in patients who received a smaller GJ. Moreover. after multivariate regression the 30 mm GJ was not identified as an independent predictor of weight loss after two years (Table 3). But in terms of all EWL categories (<25%, 25–49%, ≥50%) we revealed a statistical significance between the two groups (P value χ^2^ for trend <0.035) favoring the 30 mm sized GJ (Table 2). Thus, a lower rate of therapy failure among patients in the 30 mm sized GJ group in comparison to patients who received a 45 mm size GJ can be assumed. In addition, only patients (n = 4) in the 45 mm GJ group had even a weight gain (Table 2).

Another argument for conducting smaller sized GJs may be the aim to use less foreign materials with smaller cartridges, in order to prevent material related complications such as fistulas [24]. On the other hand, a too small GJ may lead to clinical relevant stenosis, which is a frequently published complication following LRYGB [10,25,26]. To that. Fisher et al. (2007) performed a randomized clinical trial (n = 200) on the 21 mm and 24 mm sized GJs [27]. The authors revealed a higher rate of stenosis among patients, who received a 21 mm GJWe did not diagnose a postoperative stenosis among our patients.

Weight regain following LRYGB is a common and long-term complication. Up to 30% of patients regain weight within 10 years postoperative [28]. The etiology seems to be multifactorial, but some risk factors have been defined. Increasing psychological symptoms like binge eating disorder seem to correlate with weight regain [2,6]. Moreover, some authors postulated that nutritional habit, a permissive psychosocial enviroment and patient's noncompliance may also lead to weight regain after LRYGB. Furthermore, it has been demonstrated that an increased GJ size seems to be also a risk factor for weight regain after LRYGB. Abu Dayyeh et al. revealed that a 40 mm and 50 mm sized GJs led to a more frequent weight regain than 10 mm, 20 mm and 30 mm GJ [20]. Confirming this assumption, no patients in the 30 GJ group reported weight regain. Only individuals of the 45 mm GJ (n = 4) suffered from weight regain.

We identified female gender as an independent predictor for weight loss after two years. Reviewing the literature we did not reveal any explanation on that. We assume, that women might be more disciplined in terms of postoperative nutrition recommendations.

In summary, due to our findings we assume that a 30 mm sized GJ may combine the advantages of “smaller” GJs in terms of EWL with less weight regain and the advantages of “bigger” GJs regarding less postoperative stenosis. But prospective randomized clinical trials comparing different GJ sizes, completed in a same approach, are mandatory to confirm our assumption.

As study limitations due to lack of a long-term follow-up we are not able to determine the rate and extent of long-term EWL and weight regain among our patients. The GJs among our patients were only completed with a linear stapler. We did not investigate the impact of anastomosis technique. Moreover, the pouch volumes were estimated and not exactly measured. In addition, the groups differ with significance in terms of that (Table 1). But multivariate regression did not identify pouch volume as an independent predictor for weight loss after two years (Table 3). Missing of patients ASA-scoring also must be mentioned. As an elective surgical procedure among relatively young patients (41.7 ± 12.3 years) the great majority of operated patients had an ASA-score of II. Furthermore, the operating time and the length of hospital stay (in general 3 days after surgery) were not documented. Due to the anonymized data set these information's could not be extracted afterwards. As another bias, in terms of baseline characteristics, we revealed a higher BMI at time of operation in the “30 mm sized GJ group” (Table 1). Most likely this selection bias is explained by surgeon's attitude. The individuals with highest BMI were maybe operated on by the more experienced surgeon, who preferred a 30 mm sized GJ. Moreover, It has been published in literature and confirmed in our multivariate regression (Table 3), that a higher preoperative BMI may lead to a more sufficient weight loss postoperative [29]. As further study limitations, the follow-up appointments were irregularly. In some cases, the patients did not show up.

## 6 Conclusion

A 30 mm sized gastrojejunostomy may lead to a lower rate of therapy failure in comparison to a 45 mm sized gastrojejunostomy following laparoscopic Roux-en-Y gastric bypass. Prospective randomized trials are mandatory to confirm our findings.

## Declaration of competing interest

All authors declare that they have no conflict of interest.

## Informed consent

Informed consent was not obtained.

## Provenance and peer review

Not commissioned, externally peer-reviewed.

## Ethical approval

In accordance with the ethical standards of the Helsinki Declaration of 1975 the trial has been ethically approved in December 2020 by the Ethics Committee Ärztekammer Berlin (Medical Association Berlin) in October 2019 (Eth-17/19).

## Sources of funding

We acknowledge funding by the MHB Open Access Publication Fund supported by the 10.13039/501100001659German Research Association (DFG).

## Author contribution

Dr. med. Oliver Stumpf: Contribution to the paper: author, data collection, data analysis and interpretation, writing the paper, examination and treatment of the patient. Prof. Volker Lange: Examination and treatment of the patient. Dr. med. Anke Rosenthal: Examination and treatment of the patient. Prof. Rolf Lefering. Statistical analysis, writing the paper. PD Dr. med. Christoph Paasch (corresponding author): Contribution to the paper: author, data collection, data analysis and interpretation, writing the paper, examination and treatment of the patient.

## Guarantor

PD Dr. med. Christoph Paasch.

## Declaration of competing interest

None.
